# Broad Ligament Hernia Causing Small Bowel Obstruction: When Gynecologic Symptoms Conceal Surgical Emergencies—A Case Report

**DOI:** 10.1155/crog/3055029

**Published:** 2026-06-08

**Authors:** Tiffany Chang, Bella Swan, Antoun Al Khabbaz

**Affiliations:** ^1^ Department of Obstetrics and Gynecology, University of Illinois College of Medicine Rockford, Rockford, Illinois, USA; ^2^ Women′s Health Department, Crusader Community Health Clinics, Rockford, Illinois, USA

**Keywords:** broad ligament hernia, case report, ileal resection, internal hernia, small bowel obstruction

## Abstract

Internal hernias are an uncommon cause of small bowel obstruction (SBO), accounting for approximately 1% of cases. Among these, broad ligament hernias represent a rare subtype, comprising approximately 4% of internal hernias. Despite their rarity, they carry a significant risk of bowel strangulation and ischemia, necessitating prompt recognition and surgical intervention. We report the case of a 35‐year‐old G5P3023 woman with no prior abdominal surgeries who presented with a 2‐day history of sharp abdominal pain, nausea, and vomiting. Initial imaging findings were suggestive of a tubo‐ovarian abscess (TOA); the patient was managed with broad‐spectrum antibiotics and subsequently underwent an unsuccessful image‐guided drainage attempt. Due to clinical deterioration, a diagnostic laparoscopy was performed, revealing a broad ligament hernia with incarcerated small bowel. The procedure was converted to an exploratory laparotomy, during which the ischemic ileum was resected and a primary was anastomosis performed. The broad ligament defect was subsequently repaired. This case highlights the diagnostic challenges associated with internal hernias, particularly when presentation mimics gynecologic pathology. Failure to include internal hernias in the differential diagnosis may delay appropriate intervention. Early recognition based on clinical and radiologic findings is critical to improving outcomes and preventing bowel compromise.

## 1. Introduction

Internal hernias are defined as acute or chronic protrusion of intestines or other abdominal organs through a peritoneal or mesenteric opening. They are a rare cause of small bowel obstruction (SBO), accounting for approximately 1% of all obstructive cases, and are associated with a mortality rate exceeding 50% if strangulation is present [1]. They are thought to be caused by congenital mesenteric defects, abnormal embryonic development, trauma, or surgical intervention ([2]). A well‐accepted classification of internal hernias devised by Ghahremani separates internal abdominal hernias into six main groups: paraduodenal (50%–55%), foramen of Winslow (6%–10%), transmesenteric (8%–10%), pericecal (10%–15%), intersigmoid (4%–8%), and paravesical (< 4%) [3]. Broad ligament hernias are classified as paravesical, the most uncommon subtype of internal hernias. They are more commonly observed in older patients, involving the ileal segments ([2]). Although rare, broad ligament hernias carry a significant risk of bowel strangulation and ischemia, necessitating prompt recognition and surgical management. Diagnosis of internal hernias is challenging due to their nonspecific clinical presentation and the rarity of the condition, which can make characteristic imaging findings less readily recognizable. We describe a case of an uncommon subtype of primary internal hernia in a 35‐year‐old woman with no history of abdominal surgery.

### 1.1. Patient Information

We report the case of a 35‐year‐old G5P3023 woman with no prior abdominal surgeries who presented with a 2‐day history of sharp abdominal pain, nausea, vomiting, and decreased appetite. She denied diarrhea, hematochezia, melena, fever, chills, dysuria, and constipation; her last bowel movement occurred the morning of her presentation. She reported 2 days of atypical vaginal spotting. Her pain was not relieved by ibuprofen.

Her medical history was significant for hypertension (previously treated with ACE inhibitors and diuretics, not currently taking), pulmonary embolism in 2017 (treated with rivaroxaban for 1 year, no current anticoagulation), and asthma. Surgical history included dilation and curettage and diagnostic paracentesis in 2022. She denied a history of sexually transmitted infections. Family history was notable for diabetes mellitus and hypertension in her mother and stroke in her maternal grandmother.

She was sexually active, used condoms for contraception, and had a remote smoking history (0.3 pack/day, quit 10 years prior). She denied alcohol and illicit drug use.

### 1.2. Clinical Findings

On presentation to the emergency department, the patient was afebrile (97.9°F) with markedly elevated blood pressure (189/125 mmHg), had a heart rate of 90 beats per minute, respiratory rate of 19 breaths per minute, and oxygen saturation of 99% on room air. She appeared mildly distressed but otherwise well‐appearing, alert, oriented, and cooperative, without signs of toxicity or diaphoresis. Abdominal examination revealed a soft abdomen with voluntary guarding, most pronounced in the left lower quadrant, and hypoactive bowel sounds. There was no costovertebral angle tenderness bilaterally.

Genitourinary examination was notable for a small amount of old blood in the vaginal vault with foul odor, cervical motion tenderness, and left adnexal tenderness. A digital rectal examination was not documented. Initial laboratory evaluation demonstrated a negative urine pregnancy test. Urinalysis was positive for nitrites and leukocyte esterase, and urine culture grew >100,000 CFU/mL *Escherichia coli*. Complete blood count and comprehensive metabolic panel were within normal limits. Wet mount microscopy revealed clue cells without trichomonas or yeast and rare polymorphonuclear leukocytes, consistent with bacterial vaginosis.

On hospital day (HOD) 2, the patient continued to report moderately severe abdominal pain, which was initially localized to the left lower quadrant but later became diffuse with epigastric involvement and was controlled with intravenous hydromorphone. She also reported persistent nausea and vomiting, without passage of flatus or bowel movements.

## 2. Timeline

The patient′s hospital course is summarized as follows:

On HOD 1, she presented with abdominal pain, nausea, vomiting, and vaginal spotting. Imaging, including contrast‐enhanced computed tomography (CT) of the abdomen and pelvis and transabdominal pelvic ultrasound, suggested a tubo‐ovarian abscess (TOA) with possible additional abscess formation; a bowel obstruction was considered but deemed unlikely. She was started on ceftriaxone, doxycycline, and metronidazole and was admitted to the obstetrics/gynecology (OB/GYN) service, with internal medicine consultation for hypertensive management.

On HOD 2, she was managed for presumed TOA/hydrosalpinx with intravenous antibiotics and analgesia. Her blood pressure remained elevated, requiring initiation of amlodipine, hydrochlorothiazide, lisinopril, and as‐needed hydralazine, attributed in part to uncontrolled pain.

**Figure 1:**
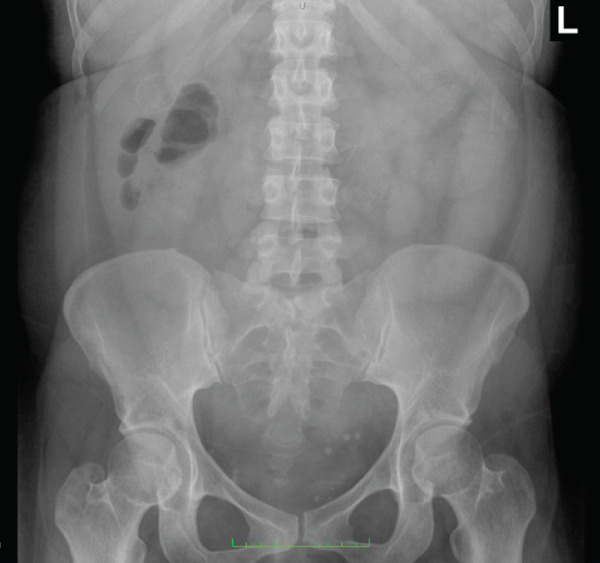
Abdominal radiograph taken on HOD 2 showing no dilated loops of bowel, indicating no clear evidence of an obstructive process at this time.

**Figure 2:**
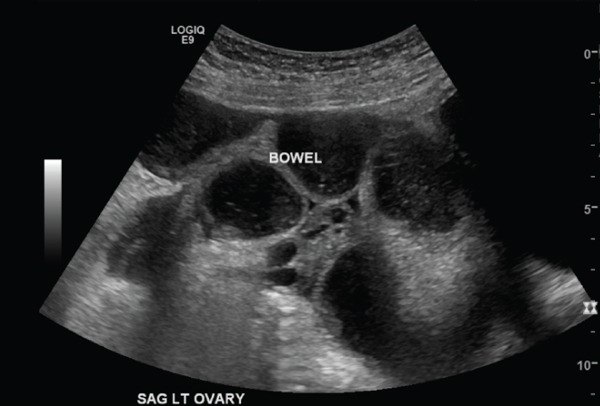
Repeat pelvic ultrasound on HOD 3 showing dilated loops of bowel, consistent with a possible obstructive process.

**Figure 3:**
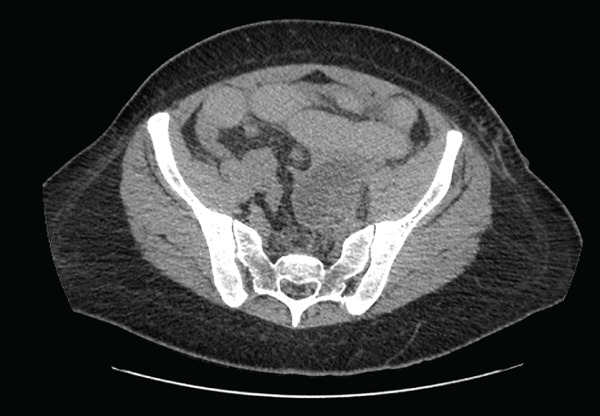
CT image from HOD 3 showing dilated loops of bowel, identified cystic process, and ascites.

By HOD 3, her pain had become diffuse and epigastric, accompanied by persistent nausea, vomiting, and absence of flatus or bowel movements. She was made nil per os, a nasogastric tube was placed, and repeat imaging was obtained (see Figure 1 and Figure 2). Interventional radiology (IR) attempted drainage of the presumed abscess without success; ascitic fluid was obtained. Subsequent CT imaging (see Figure 3) demonstrated multiple dilated bowel loops with a transition point adjacent to a left pelvic cystic structure, concerning for SBO. Surgical evaluation was obtained, and operative management was planned.

**Figure 4:**
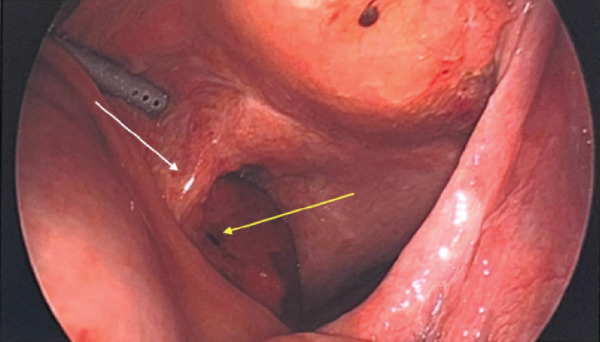
Laparoscopic view of the left‐sided broad ligament incarcerated hernia (yellow arrow) seen lateral to the uterosacral ligament (white arrow).

**Figure 5:**
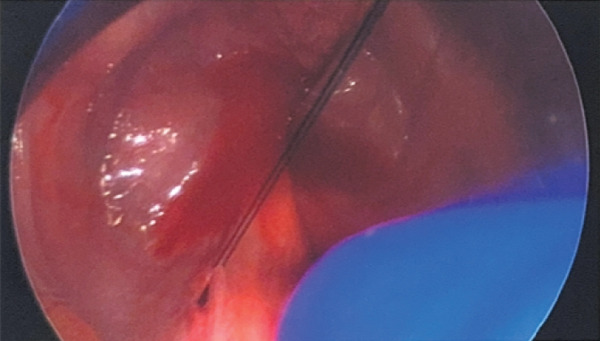
Intraoperative image capture of broad ligament defect.

**Figure 6:**
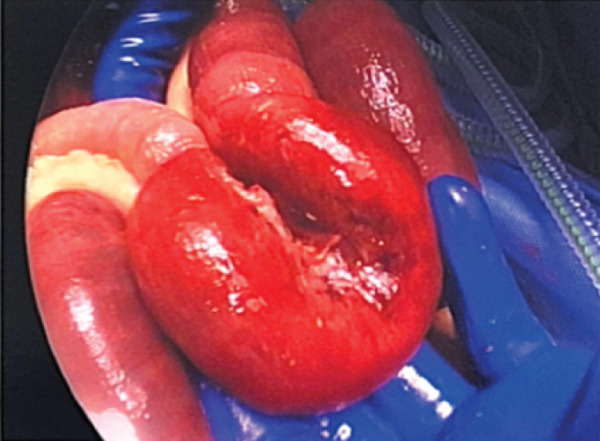
Evisceration of the small bowel during exploratory laparotomy revealed an ischemic and hemorrhagic ileal segment, noted after manual reduction of the hernia.

On HOD 4, diagnostic laparoscopy revealed dilated small bowel and an incarcerated internal hernia through a defect in the left broad ligament (see Figure 4 and Figure 5). The procedure was converted to an exploratory laparotomy with small bowel resection and primary anastomosis (see Figure 6).

Postoperatively (HODs 5–8), the patient demonstrated gradual clinical improvement. Her pain was controlled, bowel function returned with passage of flatus by postoperative day 2, and diet was progressively advanced. The nasogastric tube was removed, and she transitioned to oral analgesia. The initial diagnosis of TOA was revised, with intraoperative findings confirming SBO secondary to a broad ligament hernia; the uterus, fallopian tubes, and ovaries appeared grossly normal aside from minor endometriotic implants. She was discharged home on postoperative day 4 with appropriate medications and follow‐up.

### 2.1. Diagnostic Assessment

On presentation to the emergency department, the patient underwent a contrast‐enhanced CT scan of the abdomen and pelvis for evaluation of abdominal pain.

Imaging revealed a multilocular structure within the left adnexa and moderate free fluid in the pelvis, raising concern for a TOA. Radiology also noted a possible isolated loop of obstructed bowel, though suspicion was documented as unlikely given its deep pelvic location.

HOD 1: CT abdomen/pelvis w/IV contrast

**Figure 7:**
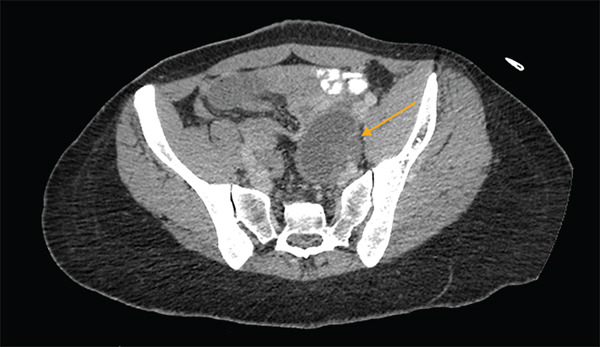
CT scan of abdomen/pelvis with IV contrast performed on admission to the emergency department on HOD 1.

1. In the left adnexa, there is a multilocular tubular structure with moderate free fluid in the pelvis that is suspicious for underlying TOA (see orange arrow in Figure 7). Additionally, extending superiorly from this tubular structure in the pelvis is an elongated air–fluid collection, which is suspicious for an additional abscess. Other etiology could potentially include an isolated loop of obstructed bowel; however, this is unlikely given the deep pelvic location. Delayed abdominal radiographs should be considered to ensure the enteric contrast is reaching the rectum. Further gynecologic evaluation should be considered. Additional pelvic ultrasound or MRI could be considered for further characterization.
2. Cholelithiasis.
3. The imaged appendix appears normal caliber and is partially air‐filled.
4. Probably small hepatic cyst.
5. Small hiatal hernia.
6. The kidneys demonstrate several heterogeneous hypodensities, the largest seen in the upper pole of the right kidney measuring 1.4 cm in size; findings could represent a complex cyst; however, these are incompletely assessed on single‐phase CT, and nonemergent renal ultrasound is recommended to evaluate the internal features.

A transabdominal ultrasound was subsequently performed, again suggesting TOA based on a fluid‐filled tubular structure with mild internal debris and adjacent inflammatory changes near the left ovary. Two days later, a pelvic ultrasound demonstrated a persistent tubular, fluid‐filled structure within the left adnexa, broadening the differential to include a dilated fallopian tube. Additionally, dilated, fluid‐filled small bowel loops were visualized, prompting recommendation for correlation with clinical signs of obstruction.

HOD 1: Pelvic US, transabdominal only

**Figure 8:**
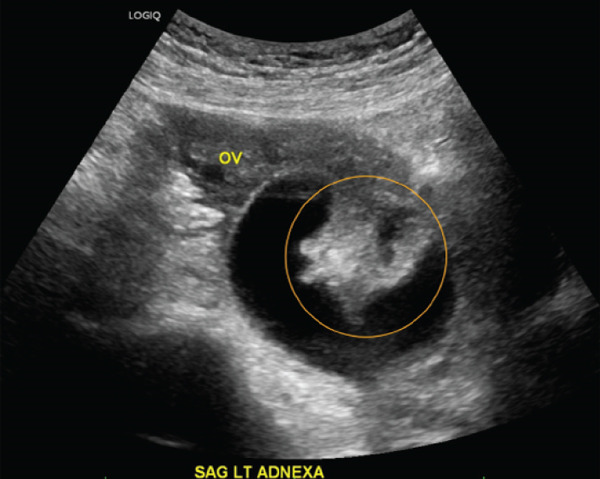
Pelvic ultrasound performed in the emergency department on HOD 1 demonstrates a “spoke and wheel” appearance, which is often associated with an abscess.

1. Limited study; the bladder is incompletely distended in transvaginal examination not performed secondary to patient pain/discomfort.
2. Normal endometrial thickness.
3. Ovaries appear normal in this study. Color duplex examination is limited secondary to factors listed above.
4. Fluid‐filled tubular structure with mild debris and suspected adjacent inflammatory changes adjacent to the left raising the possibility of TOA (see structure within orange circle in image Figure 8).
5. Moderate free fluid in the cul‐de‐sac.

HOD 3: Pelvic US

1. In the left adnexum, there is a tubular fluid‐filled structure with adjacent free fluid, which could represent a dilated fallopian tube or tubal abscess.
2. In the abdomen, more anteriorly, there are dilated and fluid‐filled loops of small bowel. Correlate with signs and symptoms of bowel obstruction.

On HOD 4, repeat CT imaging of the abdomen and pelvis without contrast showed dilated small bowel loops with a presumed transition point adjacent to the cystic process in the left hemipelvis. At this stage, the leading diagnosis was a left TOA complicated by suspected SBO.

HOD 3: CT abdomen/pelvis w/o contrast

1. Multiple dilated loops of small bowel with presumed transition point left lower adjacent to the cystic process in the left hemipelvis representing hydrosalpinx/TOA. Constellation of findings suggestive of SBO, with development of abdominopelvic extension into posterior cul‐de‐sac. Unclear whether ascites is related to obstruction versus suspected hydrosalpinx/TOA. No free air or pneumatosis. Given lack of oral contrast, recommend at least radiographic follow‐up.
2. Uncomplicated cholelithiasis.
3. No urolithiasis.
4. Remaining findings are stable from examination on 1/25/2022.

### 2.2. Therapeutic Intervention

At initial presentation, the patient was started on broad‐spectrum antibiotics with ceftriaxone, doxycycline, and metronidazole to empirically treat a suspected TOA and bacterial vaginosis. The IR team was consulted for image‐guided drainage of the presumed abscess; however, the attempt was unsuccessful. IR suggested obtaining a repeat CT abdomen/pelvis at this time and continuing with antibiotics as the diagnosis was still presumed to be a TOA.

Over the next 1–2 days, the patient′s condition deteriorated, with worsening abdominal pain, nausea, vomiting, and obstipation. Repeat CT abdomen/pelvis with contrast demonstrated progressively dilated small bowel loops with a transition point, concerning for a SBO. A nasogastric tube was placed for decompression, and the general surgery team was consulted. They reviewed the imaging and concurred with the working diagnosis of TOA, deferring surgical intervention to the OB/GYN team.

The following day, the patient was taken to the operating room by the OB/GYN team for diagnostic laparoscopy, with a preoperative diagnosis of left TOA and suspected bowel obstruction. Upon entry into the abdominal cavity, dilated loops of small bowel were encountered. After gently mobilizing the bowel away from the posterior cul‐de‐sac, a broad ligament hernia with incarcerated small bowel was identified.

The general surgery team was urgently consulted intraoperatively. The procedure was converted to an exploratory laparotomy. The general surgery team eviscerated the small bowel (see Figure 6), located and manually reduced the internal hernia, closed the defect in the broad ligament with silk suture (see Figure 5), resected approximately 20 cm of focally hemorrhagic and necrotic bowel, and performed a side‐to‐side small bowel anastomosis. Indocyanine Green (ICG) dye and the Stryker SPY fluorescence imaging camera were used to assess adequate arterial blood flow. Inflow at this time appeared evenly distributed.

### 2.3. Follow‐Up and Outcomes

Pathologic evaluation of the resected small bowel specimen (19.5 cm in length, 2.0–2.5 cm in diameter) demonstrated benign small bowel with transmural necrosis, acute inflammation, hemorrhage, and serositis, consistent with incarceration; surgical margins were viable.

At 2‐week follow‐up with the general surgery team, the patient reported intermittent gastrointestinal upset and diarrhea, attributed to prior hemorrhagic bowel changes, and was otherwise recovering appropriately. She was advised to discontinue stool softeners. At 4 weeks, she endorsed persistent abdominal discomfort with gas and bloating; repeat CT of the abdomen and pelvis with contrast was recommended but not obtained. Laboratory evaluation, including complete blood count and metabolic panel, was unremarkable aside from chronic anemia (unchanged from baseline), hypokalemia (potassium 3.0 mmol/L), and mildly elevated transaminases (AST 53 U/L, ALT 44 U/L). No additional follow‐up was documented, and the patient had no hospital readmissions.

Approximately 1 year later, she presented to the OB/GYN outpatient clinic at 15 weeks gestation and subsequently underwent an elective termination at 17 weeks gestation at an outside facility. She had no gastrointestinal concerns noted at that time.

## 3. Discussion

Preoperative suspicion for internal hernias remains challenging due to nonspecific clinical presentation. Despite their high morbidity and mortality, internal hernias remain underdiagnosed. Although once considered a rare cause of SBO, internal hernias are increasingly recognized with the rise of transmesenteric, transmesocolic, and retroanastomotic procedures such as bariatric surgery and liver transplantation ([1]). While prior abdominal surgery is a well‐established risk factor for hernia formation, the association between broad ligament defects and surgical history appears less consistent, with multiple cases reported in patients without prior abdominal or pelvic surgery, suggesting a congenital or spontaneous etiology [4].

Distinguishing TOA from entrapped bowel on imaging is critical to timely management. TOA classically presents as a complex, rim‐enhancing adnexal mass with a thick, enhancing abscess wall, often with associated inflammatory changes and systemic symptoms such as fever and leukocytosis [5]. In contrast, broad ligament hernias demonstrate clustered dilated small bowel loops lateral to the uterus, convergence of engorged vessels at the defect, and displacement of pelvic organs such as the rectosigmoid colon and uterus—typically anterior deviation of the uterus and posterior‐lateral displacement of the rectosigmoid colon [6]. An increased distance between the uterus and ipsilateral ovary in opposite directions may further suggest a hernia rather than an adnexal abscess [6]. In general, the appearance of dilated loops or a “sac‐like appearance” of bowel loops at an abnormal anatomic location is highly suggestive of an internal hernia causing a closed‐loop obstruction, and the patient should be evaluated with this diagnosis in mind [7].

Similar to our case, several published reports describe an inability to diagnose internal hernia prior to surgical intervention, which supports the need for improved recognition of characteristic imaging findings to establish an accurate preoperative diagnosis [7, 8]. In patients presenting with acute pelvic pain and imaging suggestive of gynecologic pathology with concomitant bowel obstruction, early involvement of the general surgery team should be considered. Diagnostic laparoscopy is often warranted when clinical findings are atypical for TOA or when there is clinical concern for an evolving obstruction. In our case, recognition of the patient′s atypical presentation for TOA and correlation with imaging findings warranted prompt surgical evaluation to prevent bowel strangulation and ischemia. IR drainage was attempted prior to proceeding with surgical intervention given the patient′s lack of response to antibiotics, worsening symptoms, and a prematurely narrowed differential diagnosis that did not include internal hernia.

The suggested diagnostic approach to CT imaging analysis includes detection of an intestinal closed loop, identification of the hernia orifice, and appreciation of abnormal displacement of surrounding structures and mesenteric vessels using intravenous contrast material [9]. Therefore, radiologists play an important role in initial detection of internal hernias, which can aid in more prompt and accurate preoperative diagnosis, leading to appropriate surgical management and therefore improved patient outcomes.

In this case, the initial radiologic suggestion of TOA in combination with insufficient suspicion for internal hernias contributed to a narrowed differential diagnosis despite the absence of fever or leukocytosis and the presence of small bowel dilation. Untimely recognition of these characteristic imaging findings resulted in delayed intervention and increased risk of a closed‐loop bowel obstruction and therefore bowel compromise, requiring conversion from a minimally invasive laparoscopy by the OB/GYN team to an exploratory laparotomy by the general surgery team.

## 4. Conclusion

Internal hernias should remain on the differential diagnosis of SBO, even in patients without prior surgical history and in cases initially suggestive of gynecologic pathology. Early recognition and intervention are essential to prevent bowel compromise and improve patient outcomes.

## Author Contributions

Tiffany Chang: formal analysis, investigation, writing—original draft, and writing—review & editing. Bella Swan: formal analysis, investigation, writing—original draft, and writing—review & editing. Antoun Al Khabbaz: funding acquisition, resources, project administration, and supervision.

## Funding

This study was supported by the Department of Obstetrics and Gynecology, University of Illinois College of Medicine Rockford.

## Consent

Written informed consent was obtained from the patient for publication and use of intraoperative images and radiological images in this case report. All identifiable patient information has been removed.

## Conflicts of Interest

The authors declare no conflicts of interest.

## Supporting information


**Supporting Information** Additional supporting information can be found online in the Supporting Information section. File S1: CARE checklist (English, 2013): Completed CARE (CAse REport) guideline checklist corresponding to this case report. This case report was prepared in accordance with the CARE reporting guidelines, and the completed CARE checklist is provided as supporting information (File S1).

## Data Availability

Data sharing is not applicable to this article as no datasets were generated or analyzed during the current study.
