# Supplementary material for: Broad Ligament Hernia Causing Small Bowel Obstruction: When Gynecologic Symptoms Conceal Surgical Emergencies—A Case Report
Source: Case Rep Obstet Gynecol. 2026 Jun 8;2026:3055029. doi: 10.1155/crog/3055029 (PMC13244381; doi:10.1155/crog/3055029)
Supplement: Supplementary file 1 — Supporting Information Additional supporting information can be found online in the Supporting Information section. File S1: CARE checklist (English, 2013): Completed CARE (CAse REport) guideline checklist corresponding to this case report. This case report was prepared in accordance with the CARE reporting guidelines, and the completed CARE checklist is provided as supporting information (File S1). [file CROG-2026-3055029-s001.pdf]

CARE
case report guidelines

CARE Checklist of information to include when writing a case report

(cc) BY-NC-ND

Topic	Item	Checklist item description	Reported on Line
**Title**	**1**	The diagnosis or intervention of primary focus followed by the words "case report"	1-2
**Key Words**	**2**	2 to 5 key words that identify diagnoses or interventions in this case report, including "case report"	30-31
**Abstract (no references)**	**3a**	Introduction: What is unique about this case and what does it add to the scientific literature?	33-37, 47-51
	**3b**	Main symptoms and/or important clinical findings	38-39
	**3c**	The main diagnoses, therapeutic interventions, and outcomes	40-46
	**3d**	Conclusion—What is the main "take-away" lesson(s) from this case?	45-49
**Introduction**	**4**	One or two paragraphs summarizing why this case is unique (**may include references**)	47-51
**Patient Information**	**5a**	De-identified patient specific information.	73
	**5b**	Primary concerns and symptoms of the patient.	74-78
	**5c**	Medical, family, and psycho-social history including relevant genetic information	79-86
	**5d**	Relevant past interventions with outcomes	82
**Clinical Findings**	**6**	Describe significant physical examination (PE) and important clinical findings.	88-103
**Timeline**	**7**	Historical and current information from this episode of care organized as a timeline	109-139
**Diagnostic Assessment**	**8a**	Diagnostic testing (such as PE, laboratory testing, imaging, surveys).	142-223
	**8b**	Diagnostic challenges (such as access to testing, financial, or cultural)	n/a
	**8c**	Diagnosis (including other diagnoses considered)	207-208
	**8d**	Prognosis (such as staging in oncology) where applicable	n/a
**Therapeutic Intervention**	**9a**	Types of therapeutic intervention (such as pharmacologic, surgical, preventive, self-care)	226-231, 235
	**9b**	Administration of therapeutic intervention (such as dosage, strength, duration)	n/a
	**9c**	Changes in therapeutic intervention (with rationale)	240-250
**Follow-up and Outcomes**	**10a**	Clinician and patient-assessed outcomes (if available)	260-266
	**10b**	Important follow-up diagnostic and other test results	267-272
	**10c**	Intervention adherence and tolerability (How was this assessed?)	272
	**10d**	Adverse and unanticipated events	n/a
**Discussion**	**11a**	A scientific discussion of the strengths AND limitations associated with this case report	277-331
	**11b**	Discussion of the relevant medical literature **with references**.	277-306, 317-320
	**11c**	The scientific rationale for any conclusions (including assessment of possible causes)	306-315, 320-323
	**11d**	The primary "take-away" lessons of this case report (without references) in a one paragraph conclusion	325-331
**Patient Perspective**	**12**	The patient should share their perspective in one to two paragraphs on the treatment(s) they received	n/a
**Informed Consent**	**13**	Did the patient give informed consent? Please provide if requested	**Yes** [x] **No** [ ]
